# Evidence of association of circulating epigenetic-sensitive biomarkers with suspected coronary heart disease evaluated by Cardiac Computed Tomography

**DOI:** 10.1371/journal.pone.0210909

**Published:** 2019-01-23

**Authors:** Teresa Infante, Ernesto Forte, Concetta Schiano, Bruna Punzo, Filippo Cademartiri, Carlo Cavaliere, Marco Salvatore, Claudio Napoli

**Affiliations:** 1 IRCCS SDN, Naples, Italy; 2 Department of Medical, Surgical, Neurological, Metabolic and Geriatric Sciences, Università degli Studi della Campania ‘Luigi Vanvitelli’, Naples, Italy; Universiteit van Amsterdam, NETHERLANDS

## Abstract

Circulating biomarkers available in clinical practice do not allow to stratify patients with coronary heart disease (CHD) prior the onset of a clinically relevant event. We evaluated the methylation status of specific genomic segments and gene expression in peripheral blood of patients undergoing Cardiac Computed Tomography (CCT) for CHD (n = 95).

We choose to investigate cholesterol metabolism. Methylation and gene expression of low density lipoprotein receptor (LDLR), sterol regulatory element-binding factor 2 (SREBF2) and ATP-binding cassette transporter 1 (ABCA1) were evaluated by qRT-PCR. Calcium score (CACS), stenosis degree, total plaque volume (TPV), calcified plaque volume (CPV), non-calcified plaque volume (NCPV) and plaque burden (PB) were assessed in all CHD patients (n = 65). The percentage of methylation at the specific analyzed segment of LDLR promoter was higher in CHD patients vs healthy subjects (HS) (n = 30) (*p* = 0.001). LDLR, SREBF2 and ABCA1 mRNAs were up-regulated in CHD patients vs HS (*p* = 0.02; *p* = 0.019; *p* = 0.008). SREBF2 was overexpressed in patients with coronary stenosis ≥50% vs subjects with stenosis <50% (*p* = 0.036). After adjustment for risk factors and clinical features, ABCA1 (*p* = 0.005) and SREBF2 (*p* = 0.010) gene expression were identified as independent predictors of CHD and severity. ROC curve analysis revealed a good performance of ABCA1 on predicting CHD (AUC = 0.768; *p*<0.001) and of SREBF2 for the prediction of disease severity (AUC = 0.815; *p*<0.001). Moreover, adjusted multivariate analysis demonstrated SREBF2 as independent predictor of CPV, NCPV and TPV (*p* = 0.022; *p* = 0.002 and *p* = 0.006) and ABCA1 as independent predictor of NCPV and TPV (*p* = 0.002 and *p* = 0.013).

CHD presence and characteristics are related to selected circulating transcriptional and epigenetic-sensitive biomarkers linked to cholesterol pathway. More extensive analysis of CHD phenotypes and circulating biomarkers might improve and personalize cardiovascular risk stratification in the clinical settings.

## Introduction

Despite advances in diagnosis, treatment and prognosis, coronary heart disease (CHD) is still the most prevalent cause of mortality and morbidity worldwide [1].

The main pathophysiological process underlying the development of CHD is represented by coronary atherosclerosis whose pathogenesis involves an imbalanced lipid metabolism and impaired immune response. These phenomena contribute to endothelial dysfunction and chronic inflammation, with the consequent formation of the atherosclerotic plaque, erosion and unstable atheroma, and vessel lumen stenosis [2,3].

Several studies reported an implication of epigenetic modifications in the pathogenesis of multifactorial diseases such as CHD, focusing on the evaluation of global DNA methylation in atherosclerotic tissues, and in peripheral blood cells of CHD patients [4–9].

Blood gene expression profiling showed a differential transcriptional signature in CHD patients and healthy subjects (HS). Major alterations were detected in genes coding for biomolecules involved in oxidative stress, cell motility, metabolic pathways, and inflammation. Interestingly, the expression pattern was found to correlate with the severity of CHD and gene expression in vascular tissues, suggesting a mirroring between circulating cells and changes in the atherosclerotic coronary wall [10–14].

Lipid homeostasis plays a key role in the atherosclerotic process and genes actively involved are represented by low-density lipoprotein receptor (LDLR) that has a central role in regulating the internalization of plasma LDL-cholesterol (LDL-c); sterol regulatory element binding transcription factor 2 (SREBF2), involved at transcriptional level in cholesterol metabolism, and ATP binding cassette transporter1 (ABCA1), the main regulator of cholesterol cellular efflux [15–18].

Few studies have correlated circulating molecular patterns to quantitative imaging parameters derived by Cardiac Computed Tomography (CCT) [19–23].

CCT is a powerful diagnostic tool to rule out CHD thanks to its high negative predictive value [24–26] allowing characterization and quantification of atherosclerotic plaque burden (PB) and providing comprehensive information about the location, severity and features of coronary atherosclerotic plaques [27,28]. Although CCT represents the most promising tool for CHD assessment, mainly to avoid unnecessary invasive coronary angiography, it is challenging to improve the conventional risk scores by assessing new non-invasive biomarkers that could support patient stratification and clinical decision making toward personalized treatments.

Given the central role both of the epigenetics and immune system in the pathogenesis of atherosclerosis and CHD development through dynamic changes of molecular patterns [26,29,30], in this observational study we investigated the association between gene expression/epigenetic markers and CHD features. Indeed, we evaluated by quantitative realtime PCR (qRT-PCR) the methylation status of specific genomic segments and the relative expression of genes in peripheral blood mononuclear cells (PBMNCs) of patients with suspected CHD underwent to CCT aiming to find a possible screening methodology for non-invasive and non-radiation-utilizing detection of CHD. We analyzed epigenetic-sensitive genes involved in cholesterol bioactivity such as LDLR, SREBF2 and ABCA1 in correlation with CHD features and quantitative imaging parameters derived by CCT.

## Methods

### Study population

The study has been approved by the institutional ethics committee (IRCCS Fondazione SDN, protocol no. 7–13) on research on humans in accordance with the ethical guidelines of the 1975 Declaration of Helsinki. A written informed consent was obtained from all subjects enrolled.

During the experimental design we performed a power calculation analysis using G*Power software, obtaining an estimated total sample size of 81 with an effect size equal to 0.45. In a period of 36 months, between November 2014 and November 2017, 250 consecutive patients were enrolled in the study at IRCCS SDN (Naples, Italy) (see S1 Appendix for sample characterization).

Patients with known history of cancer (n = 51), active infections (n = 9), chronic or immune-mediated diseases (n = 15) were excluded from the study to avoid confounding effects due to other variables. Furthermore, subjects with cardiomyopathy, known CHD, previous percutaneous transluminal coronary angioplasty and coronary artery bypass grafting (n = 80), systemic atherosclerosis such as lower extremity peripheral arterial disease or supra-aortic arterial disease were not included in the study population. The remaining 95 subjects without a history of cardiovascular events and referred to our institution for suspected CHD were included in the study.

In detail, patients with Calcium Score (CACS) = 0 and uninjured coronaries were considered as HS (n = 30). Obstructive CHD was defined by the presence of a stenosis greater than or equal to 50% in one or more of the major coronary arteries detected by CCT.

### Sample collection and molecular analysis

PBMNCs were isolated by Ficoll gradient using HISTOPAQUE-1077 (Sigma Diagnostics, USA) according to manufacturer’s instructions and frozen at -80°C at the IRCCS SDN Biobank [31]. (S1 Appendix for details).

### Methylated DNA immunoprecipitation (MeDIP)

For DNA extraction and immunoprecipitation from isolated PBMNCs was used MagMeDIP kit (Diagenode, Belgium) (see S1 Appendix for the detailed protocol).

Genome Browser and Methbase [32] tools were used to select genetic regulatory regions and design methylation-specific primers for specific genomic sequences: LDLR promoter and intron 1, SREBF2 promoter and ABCA1 5’UTR (S1 Appendix for details).

### RNA extraction and quantitative realtime PCR assay

Total RNAs were extracted from PBMNCs of patients and HS using TRIzol solution (Thermo Fischer Scientific, USA), according to the manufacturer’s instructions (S1 Appendix for details). Oligonucleotide sequences are reported in **S1 Table**. The relative expression levels of mRNA were measured by CFX96 Touch Real-Time PCR Detection System (BioRad Laboratories, Ltd, USA). Target gene expression levels were normalized using RPS18 as housekeeping gene [33] for each sample (S1 Appendix for details). Each sample was analyzed in triplicate and data expressed as mean **±** standard error. For logistic regression analysis delta Ct (Δct) values of each gene were considered.

### Cardiac computed tomography and image analysis

**Fig 1:**
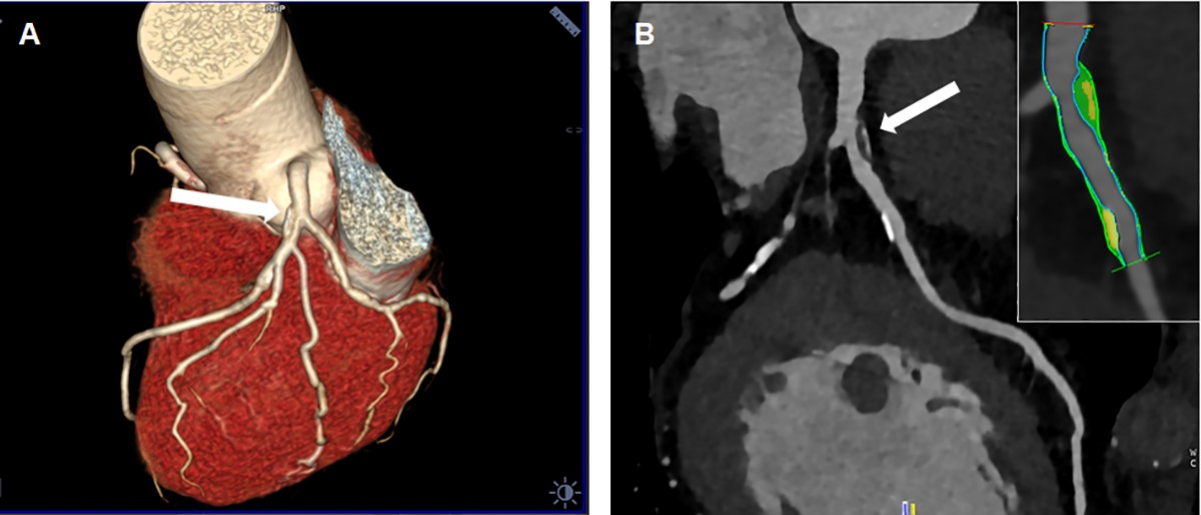
**(A)** 3D volume rendering and (**B)** Curved multi planar reconstruction of a left anterior descending artery showing a proximal mostly non-calcified plaque with a spotty calcification (white arrow in (**A)** and (**B)** panels) and a distal calcified plaque. Semi-automatic segmentation of coronary vessel: calcified component (yellow), lipid component (green) and fibrotic (blue).

**Fig 2:**
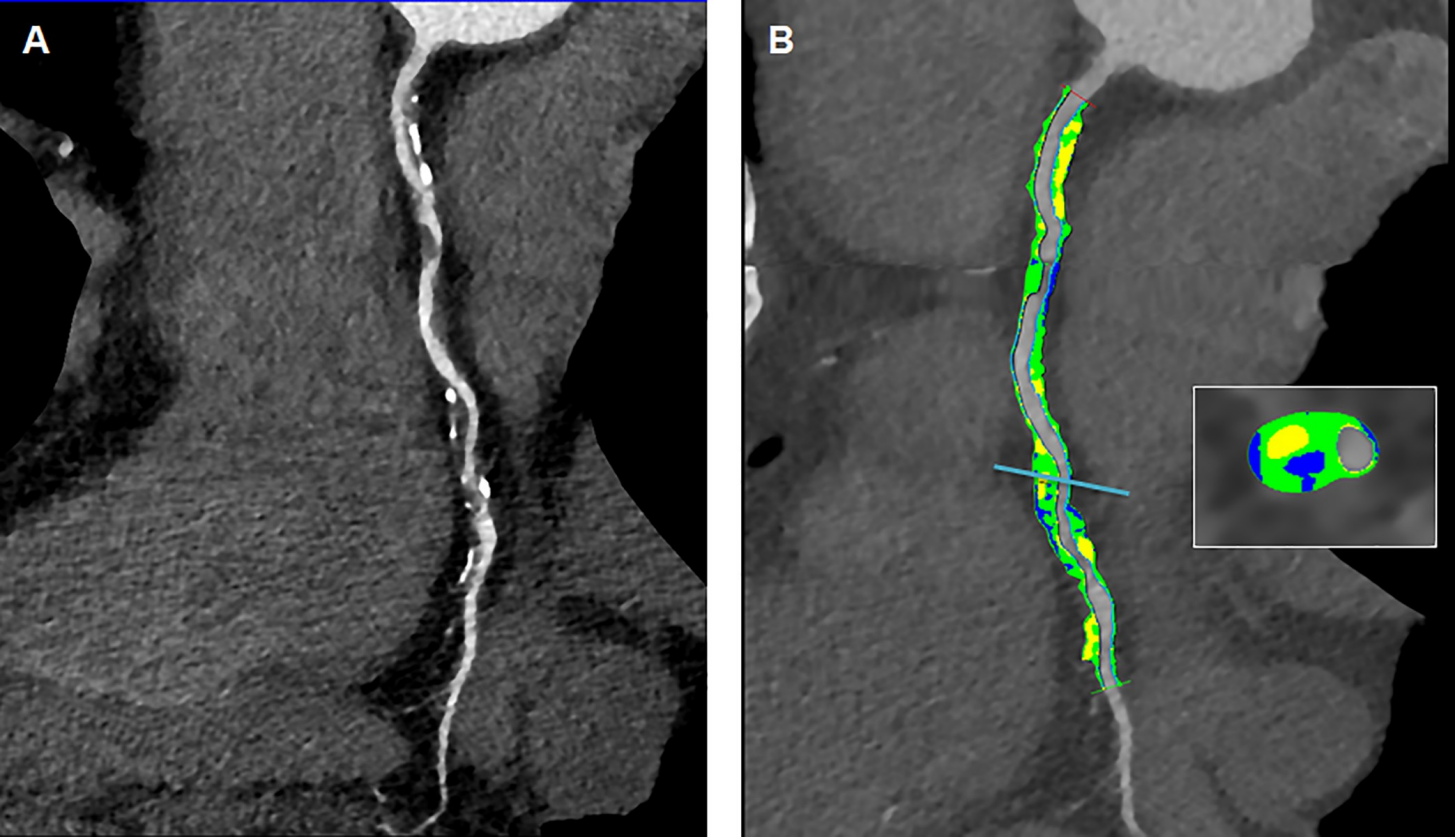
**(A)** Curved multi planar reconstruction of a right coronary artery showing a severe atherosclerosis with both calcified and non-calcified plaques. (**B)** Semi-automatic segmentation of coronary vessel: calcified component (yellow), lipid component (green) and fibrotic (blue).

All patients underwent a CCT with a third-generation dual source multidetector computed tomography scanner (Somatom Force, Siemens Healthcare AG, Germany). Details on the imaging protocols are given in the S1 Appendix. CCT derived parameters such as CACS, stenosis degree (in percentage of lumen reduction), non-calcified plaque volume (NCPV), calcified plaque volume (CPV), total plaque volume (TPV) and PB were calculated as previously reported [34]. Plaque segmentations were performed by two independent technicians and averaged values for each derived imaging parameter were considered. Only three patients showed multivessel CHD at CCT for whom the most significant plaque was considered for the segmentation and statistical analysis. All scans were analyzed by 2 experienced radiologists. After independent evaluations were made, a consensus interpretation was achieved according to the international SCCT guidelines [35]. Patients were grouped according to imaging parameters. Two representative examples of plaque segmentation by CCT to calculate imaging parameters are reported in **Fig 1** and **Fig 2**.

### Statistical analysis

Statistical analysis was performed using R Core Team (version 3.03, Austria). Continuous variables were expressed as mean ± standard deviation or standard error. Data were tested for normality through the Shapiro-Wilk test. Unpaired Student’s t-test or Mann-Whitney U test, as required, were used for comparison between two groups. The one-way analysis of variance (ANOVA) or the Kruskal-Wallis test were used for comparison among three or four groups for parametric and non-parametric variables, respectively, with Bonferroni’s adjustment for multiple comparisons. Categorical variables were expressed as percentage and were compared using the Chi-Square test or the Fisher’s exact test.

Methylation statistical analysis revealed a significance only for LDLR promoter methylation. For this reason data reporting SREBF2 and ABCA1 promoter and LDLR intron 1 methylation were not reported. Molecular markers were tested in a univariate logistic regression analysis; then, significant variables were included in a multivariate logistic regression analysis (stepwise forward model) adjusted for the traditional cardiovascular risk factors and baseline clinical features [36]. In order to assess the possible effects of dyslipidemia treatments (e.g., statins) on the molecular marker expression, we used an unpaired Student’s t-test to compare treated vs untreated subgroups. Receiver operating characteristic (ROC) curves were subsequently generated using CHD and obstructive CHD as the events. Areas under the curve (AUC) were compared for each single molecular variable and the multivariate model. For all tests a p<0.05 was considered for statistical significance.

## Results

### Study population features

The baseline characteristics of CHD patients and HS are summarized in **Table 1**. The mean age was 57.87±9.5 years in HS compared to 63.12±10.97 years in CHD patients (*p* = 0.016). The percentage of male was significantly higher in patients with CHD (75.4%) compared to HS (43.3%) (*p* = 0.002).The mean body mass index (BMI), the pericardial fat, the heart rate (HR) and the left ventricular ejection fraction (LVEF) were comparable between two groups (*p* = 0.728, *p* = 0.327, *p* = 0.152, *p* = 0.974, respectively). Cardiovascular risk factors such as CHD familiarity, smoke, hypertension, diabetes and dyslipidemia were not significantly different between HS and CHD patients (*p* = 0.759; *p* = 0.684; *p* = 0.484; *p* = 0.163; *p* = 0.125, respectively). Moreover, total cholesterol, LDL- and HDL-cholesterol plasmatic concentrations did not significantly differ among the two groups (**Table 1**).

**Table 1:** Baseline characteristics of CHD patients and HS.

Variables	CHD (n = 65)	HS (n = 30)	*p* value
Age (years)*	63.12 ± 11.0	57.87 ± 9.60	**0.016**
Men (%)	49 (75.4%)	13 (43.3%)	**0.002**
BMI (kg/m^2^)*	27.86 ± 4.52	27.40 ± 3.92	0.728
HR (bpm)	64.45 ± 12.39	69.70 ± 17.12	0.152
LVEF (%)	68.30 ± 9.34	68.21 ± 11.04	0.974
Pericardial fat (mL)*	175.33 ± 90.2	150.90 ± 74.00	0.327
CHD familiarity (%)	39 (60%)	17 (56.7%)	0.759
Smoke (%)	20 (30.8%)	8 (26.7%)	0.684
Hypertension (%)	43 (66.2%)	22 (73.3%)	0.484
Diabetes (%)	9 (13.8)	1 (3.3%)	0.163
Dyslipidemia (%)	37 (56.9%)	12 (40%)	0.125
Total cholesterol (mg/dL)*	186.4 ± 46.4	189.3 ± 38.7	0.721
LDL cholesterol (mg/dL)*	103.8 ± 36.4	117.3 ± 31.5	0.103
HDL cholesterol (mg/dL)*	53.0 ±18.6	50.3 ± 10.6	0.372
Physical activity (%)	27 (41.5%)	5 (16.7%)	**0.020**
Anti-hypertensive therapy (%)	39 (60%)	18 (60%)	1.000
Dyslipidemia treatment (%)	29 (44.6%)	5 (16.7%)	**0.007**

*Data are represented as mean ± SD. Bold values were considered statistically significant with a *p* < 0.05.

CHD, coronary artery disease; BMI, body mass index; HDL, high density lipoprotein; HR, heart rate; HS, healthy subjects; LVEF, left ventricular ejection fraction; LDL, low density lipoprotein

Hypertensive subjects were in treatment with blood-pressure lowering drugs while dyslipidemic patients were in treatment with statins. All diabetic patients were treated with oral hypoglycemic agents.

### CHD molecular features

A significantly higher methylation level in a promoter region of LDLR gene was found in PBMNCs of CHD patients (1.37%±0.25) as compared to HS (0.45%±0.09; *p* = 0.001).

**Fig 3:**
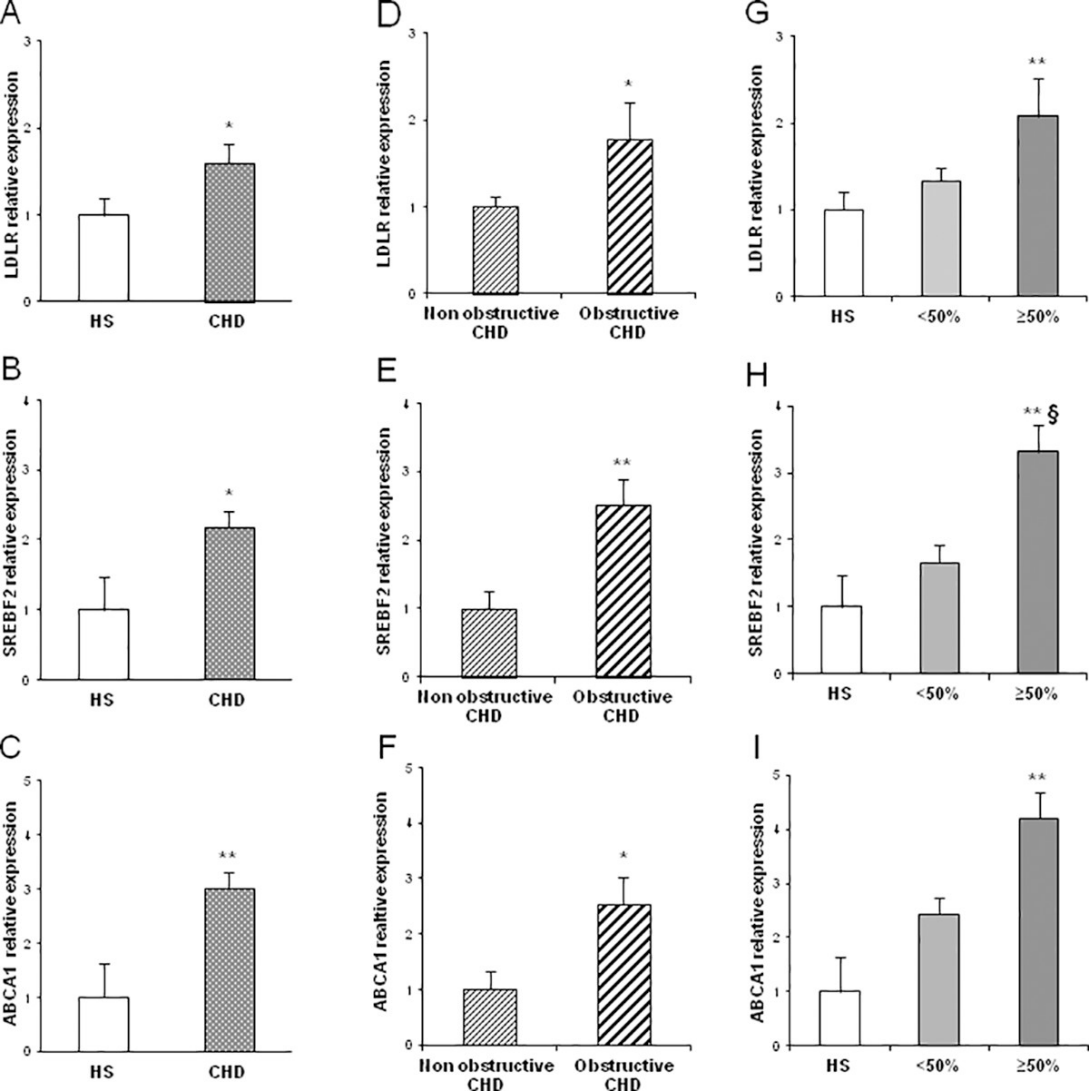
**(A-C)** LDLR, SREBF2 and ABCA1 mRNA relative expression in CHD patients (n = 65) and HS (n = 30); (**D-F)** LDLR, SREBF2 and ABCA1 mRNA relative expression in obstructive (n = 69) and non-obstructive CHD patients (n = 26); (**G-I)** LDLR, SREBF2 and ABCA1 mRNA relative expression in CHD patients with coronary stenosis <50% (n = 39) and coronary stenosis ≥50% (n = 26) compared to HS (n = 30) (*p value<0.05 vs HS; **p value<0.01 vs HS; ^§^*p* value <0.05 coronary stenosis ≥50% vs coronary stenosis <50%).

In addition, gene expression analysis showed a significant up-regulation of LDLR (*p* = 0.021), SREBF2 (*p* = 0.019) and ABCA1 (*p* = 0.008) mRNA levels in CHD patients as compared to HS (**Fig 3A, 3B** and **3C** and **Table 2**).

**Table 2:** CHD features and molecular analysis.

**CHD**	**HS**	**CHD**	***p* value**
LDLR promoter methylation**	0.45±0.09	1.37±0.25	**0.001**
LDLR gene expression^‡^	9.31±0.19	8.63±0.21	**0.021**
SREBF2 gene expression	6.94±0.48	5.81±0.23	**0.019**
ABCA1 gene expression	7.92±0.64	6.32±0.27	**0.008**
**Obstructive CHD**	**No CHD or** **CHD <50%**	**Obstructive CHD**	***p* value**
LDLR gene expression	9.07±0.12	8.24±0.43	**0.015**
SREBF2 gene expression	6.53±0.26	5.2±0.38	**0.007**
ABCA1 gene expression	7.19±0.33	5.84±0.48	**0.026**
**Stenosis degree**	**<50%**	**≥50%**	*p* value*
LDLR promoter methylation	1.3±0.15	1.47±0.2	0.708
LDLR gene expression	8.89±0.15	8.24±0.43	0.079
SREBF2 gene expression	6.21±0.28	5.21±0.39	**0.036**
ABCA1 gene expression	6.63±0.3	5.84±0.49	0.239

*p value from multiple comparisons. Bold values were considered statistically significant with a p < 0.05.

** Methylation data are expressed as percentage of methylation/total input.

^‡^Gene expression data are expressed in terms of Δct derived from qRT-PCR experiments.

ABCA1, ATP binding cassette subfamily A member 1; CHD, coronary artery disease; HS, healthy subjects; LDLR, low density lipoprotein receptor; SREBF2, sterol regulatory element-binding factor 2.

Furthermore, molecular analysis showed that higher levels of LDLR (*p* = 0.015), SREBF2 (*p* = 0.007) and ABCA1 (*p* = 0.026) were found in subjects with coronary obstructive CHD as compared to HS and patients with non-obstructive CHD. (**Fig 3D, 3E** and **3F** and **Table 2**).

Statistical differences were also found stratifying the study population according to stenosis degree. High levels of methylation were found in LDLR promoter region in patients with coronary stenosis ≥50% (1.47%± 0.20) and coronary stenosis <50% (1.3%± 0.15) compared to HS (*p* = 0.029 and *p* = 0.043, respectively) while no difference was found between CHD groups. Gene expression data demonstrated an up-regulation of LDLR (*p* = 0.007), SREBF2 (*p* = 0.003) and ABCA1 (*p* = 0.004) in CHD patients with stenosis degree ≥50% vs HS, with a significance for SREBF2 mRNA overexpression in the group with coronary stenosis ≥50% as compared to <50% (*p* = 0.036) (**Fig 3G, 3H** and **3I** and **Table 2**).

The univariate regression analysis showed that LDLR promoter methylation was significantly associated to the presence of CHD (OR = 0.464 CI 95% = 1.049–1.480; *p* = 0.043) while LDLR, SREBF2 and ABCA1 gene expression were predictors of both CHD and critical stenosis (OR = 1.516 CI95% = 1.024–2.245, *p* = 0.038; OR = 1.281 CI 95% = 1.049–1.588, *p* = 0.024; OR = 1.249 CI95% = 1.034–1.480, *p* = 0.012; respectively) (**Table 3**).

**Table 3:** Univariate and multivariate logistic regression^§^ analysis to predict CHD and obstructive CHD.

	**Univariate analysis**			
**Predictors**	**CHD**		**Obstructive CHD**	
	**OR (95% CI)**	***p* value**	**OR (95% CI)**	***p* value**
LDLR promoter methylation**	0.464 (0.220–0.976)	**0.043**	1.170 (0.921–1.485)	0.198
LDLR gene expression^‡^	1.516 (1.024–2.245)	**0.038**	0.666 (0.456–0.972)	**0.035**
SREBF2 gene expression	1.281 (1.049–1.588)	**0.024**	0.701 (0.525–0.937)	**0.016**
ABCA1 gene expression	1.249 (1.034–1.480)	**0.012**	0.806 (0.659–0.986)	**0.036**
	**Multivariate analysis**			
**Predictors**	**CHD**			
	**OR (95% CI)**			***p* value**
Male	0.121 (0.038–0.380)			<0.001
ABCA1 gene expression	1.352 (1.096–1.666)			0.005
**Predictors**	**Obstructive CHD**			
	**OR (95% CI)**			***p* value**
Male	25.757 (3.216–206.275)			0.002
SREBF2 gene expression	0.652 (0.471–0.903)			0.010

** Methylation data are expressed as percentage of methylation/total input.

^‡^Gene expression data are expressed in terms of Δct derived from qRT-PCR experiments.

^§^Multivariate analysis corrected for male gender

Bold values were considered statistically significant with a p < 0.05.

ABCA1, ATP binding cassette subfamily A member; CHD, coronary heart disease; CI, confidence interval; LDLR, low density lipoprotein receptor; OR, odds ratio; SREBF2, sterol regulatory element-binding factor 2.

**Fig 4:**
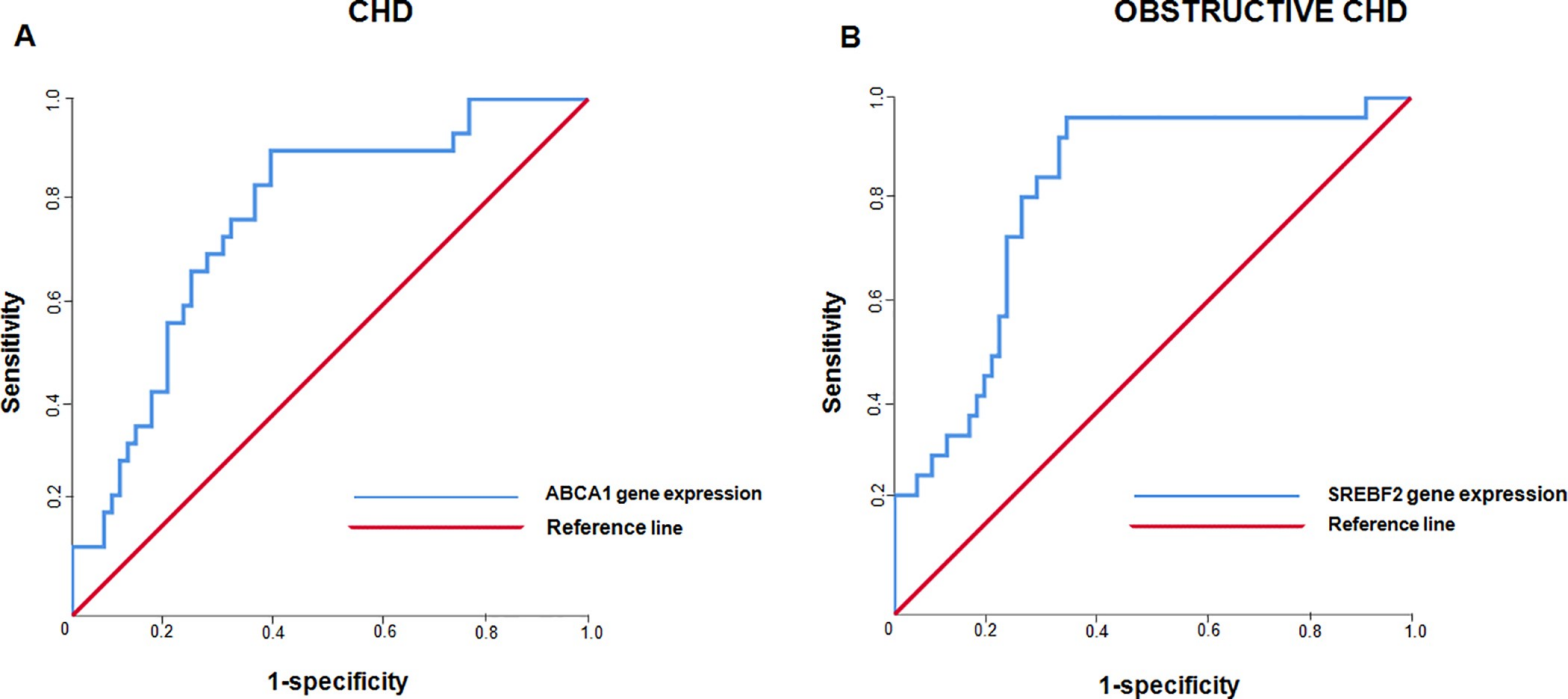
**(A)** ROC curve analysis generated from the multivariate model for the presence of CHD and ABCA1 gene expression; (**B)** ROC curve analysis generated from the multivariate model for the presence of obstructive CHD and SREBF2 gene expression.

The multivariate regression models, adjusted for cardiovascular risk factors, baseline features and clinical characteristics, showed that gender (male) and ABCA1 gene expression (OR = 1.352 CI 95% = 1.096–1.666; *p* = 0.005) were independently associated with CHD as well as SREBF2 gene expression (OR = 0.652CI 95% = 0.471–0.903; *p* = 0.010) was found to be a predictor of obstructive CHD independently by male gender (**Table 3**). ROC curve analysis of the multivariate models revealed a good performance on predicting the presence of CHD and CHD severity. In detail, ABCA1 gene expression showed an AUC of 0.768 (*p*<0.001) in predicting CHD (**Fig 4A**), while mRNA expression levels of SREBF2 provided an AUC of 0.815 (*p*<0.001) for the prediction of obstructive CHD (**Fig 4B**). Subgroup analysis comparing polygenic dyslipidemia treated vs untreated patients showed no significant differences for the expression of selected molecular markers.

### Plaque composition

According to CACS stratification (see S1 Appendix for details), molecular data showed high percentage of methylation in the analyzed CpG segment of LDLR promoter in severe group compared to normal, low and moderate groups (*p*<0.001; *p* = 0.017; *p* = 0.002, respectively) (**S1A Fig**). An up-regulation of SREBF2 and ABCA1 mRNA levels was observed in severe (*p* = 0.002 for both markers) and moderate (*p* = 0.038, and *p* = 0.008, respectively) compared to normal group. Moreover, SREBF2 gene was overexpressed in severe compared to low group (*p* = 0.038) (**S1B** and **S1C Fig**).

**Fig 5:**
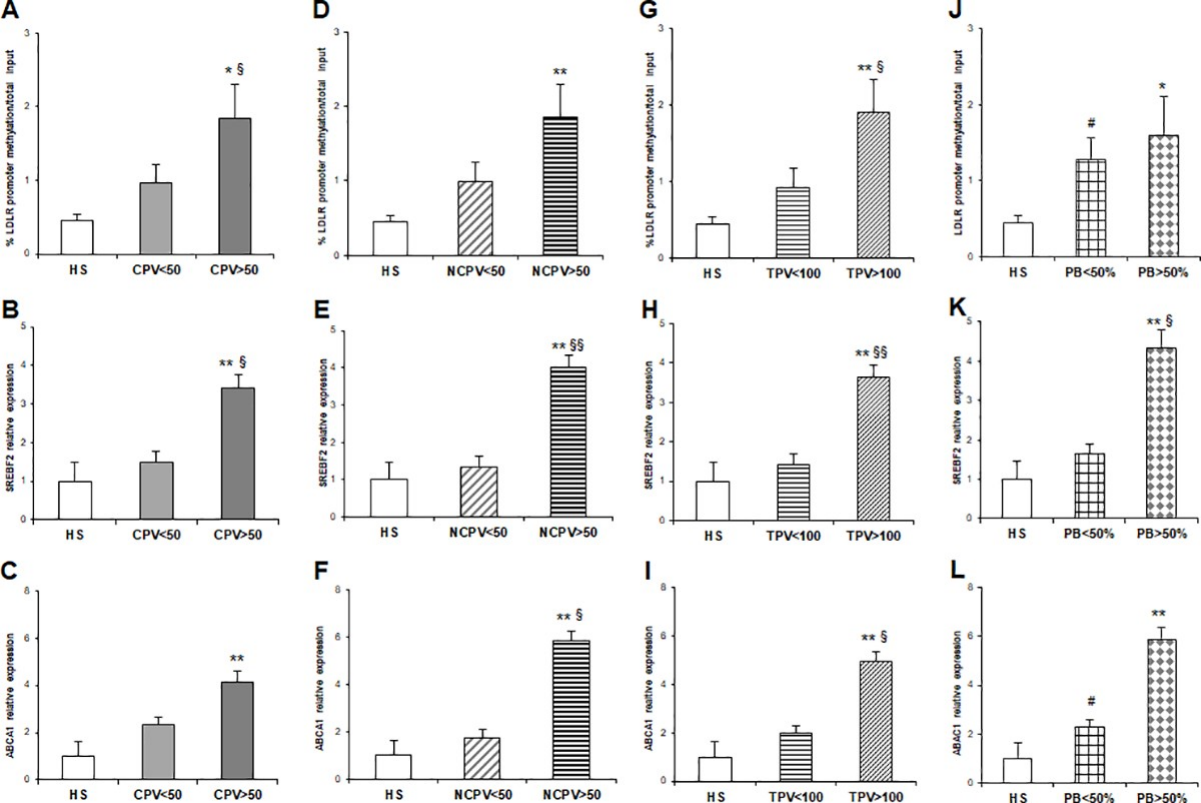
**(A)** % of LDLR promoter methylation/total input in CHD patients with CPV<50 (n = 35) and CPV>50 (n = 30) compared to HS (n = 30); (**B-C)** SREBF2 and ABCA1 mRNA relative expression in CHD patients with CPV<50 and CPV>50 vs HS; (**D)** % of LDLR promoter methylation/total input in CHD patients with NCPV<50 (n = 36) and NCPV>50 (n = 29) compared to HS (n = 30); (**E-F)** SREBF2 and ABCA1 mRNA relative expression in CHD patients with NCPV<50 and NCPV>50 vs HS; (**G)** % of LDLR promoter methylation/total input in CHD patients with TPV<100 (n = 35) and TPV>100 (n = 30) compared to HS (n = 30); (**H-I)** SREBF2 and ABCA1 mRNA relative expression in CHD patients with TPV<100and TPV>100 vs HS; (**K-L)** SREBF2 and ABCA1 mRNA relative expression in CAD patients with PB<50% (n = 46) and PB>50% (n = 19) vs HS (^§^*p* value<0.05 vs PB<50); (*p value<0.05 vs HS; **p value<0.01 vs HS for all comparisons).

As regarding quantitative plaque parameters (see Supplementary material for details), LDLR promoter methylation was higher in CHD patients with CPV>50 compared to HS (*p* = 0.02) and to CPV<50 (*p* = 0.036) (**Fig 5A**). SREBF2 mRNA was up-regulated in CPV>50 group vs HS (*p* = 0.002) and in CPV>50 vs CPV<50 group (*p* = 0.026) (**Fig 5B**). ABCA1 relative expression was higher in CPV>50 respect to HS (*p* = 0.004) with no statistically significant difference between the two CHD subgroups (**Fig 5C** and **Table 4**).

**Table 4:** Plaque composition and molecular analysis.

**NCPV (mm**^**3**^**)**	**<50**	**>50**	*p* value*
LDLR promoter methylation**	0.99±0.26	1.85±0.45	0.104
SREBF2 gene expression^**‡**^	6.51±0.28	4.94±0.33	**0.008**
ABCA1 gene expression	7.09±0.31	5.36±0.39	**0.022**
**CPV (mm**^**3**^**)**	**<50**	**>50**	*p* value*
LDLR promoter methylation	0.96±0.25	1.85±0.45	**0.036**
SREBF2 gene expression	6.36±0.29	5.17±0.35	**0.026**
ABCA1 gene expression	6.7±0.31	5.87±0.44	0.214
**TPV (mm**^**3**^**)**	**<100**	**>100**	*p* value*
LDLR promoter methylation	0.92±0.26	1.90±0.44	**0.021**
SREBF2 gene expression	6.44±0.29	5.08±0.33	0.010
ABCA1 gene expression	6.92±0.30	5.62±0.43	0.048
**PB**	**<50%**	**>50%**	*p* value*
LDLR promoter methylation	1.28±0.29	1.60±0.51	0.493
SREBF2 gene expression	6.22±0.25	4.82±0.44	**0.015**
ABCA1 gene expression	6.71±0.30	5.37±0.50	0.065

*p value from multiple comparisons. Bold values were considered statistically significant with a p < 0.05.

** Methylation data are expressed as percentage of methylation/total input.

^‡^Gene expression data are expressed in terms of Δct derived from qRT-PCR experiments.

ABCA1, ATP binding cassette subfamily A member 1; CPV, calcified plaque volume; LDLR, low density lipoprotein receptor; NCPV, non-calcified plaque volume; PB, plaque burden; SREBF2, sterol regulatory element-binding factor 2; TPV, total plaque volume.

Results from statistical analysis derived from the NCPV and the molecular data showed a differential methylation of LDLR promoter segment in CHD patients with NCPV>50 compared to HS (*p* = 0.005) but this epigenetic data was not able to discriminate patients with a prevalent non-calcified plaque composition (NCPV>50 vs NCPV<50 patients) (**Fig 5D**).

Gene expression analysis revealed an overexpression of SREBF2 and ABCA1. In detail, SREBF2 relative expression was higher in NCPV>50 patient category compared to HS (*p* = 0.001) with a differential overexpression as compared to NCPV<50 patients (*p* = 0.008). Significantly high expression levels of ABCA1 gene were found in NCPV>50 vs HS (*p* = 0.001) and NCPV>50 vs NCPV<50 (*p* = 0.022) (**Fig 5E** and **5F** and **Table 4**).

MeDIP data showed an increased methylation in LDLR promoter of CHD patients with a TPV >100 (1.9%±0.44) compared to HS (*p* = 0.001) with a significant difference between TPV >100 and TPV <100 (0.92%±0.26; *p* = 0.021) groups (**Fig 5G**). SREBF2 and ABCA1 gene expression were significantly higher in TPV>100 compared to HS (*p* = 0.001 for both genes). Furthermore, SREBF2 and ABCA1 relative expressions were significantly higher in TPV>100 group compared to TPV<100 (*p* = 0.01 and *p* = 0.048, respectively) (**Fig 5H** and **5I** and **Table 4**).

The analysis of molecular data in association to PB showed that LDLR promoter methylation, and the relative expression of SREBF2 and ABCA1 genes were overall associated with PB >50%. In particular, the percentage of methylation at the analyzed locus in LDLR promoter was high in CHD patients with PB>50% (1.60%±0.51; *p* = 0.024) compared to HS and in PB<50% patients (1.28%±0.29; *p* = 0.042) compared to HS. No statistical difference was found between the two CHD groups (**Fig 5J**).

SREBF2 gene was significantly upregulated in PB>50% patients compared to HS and to PB<50 patients (*p* = 0.001 and *p* = 0.015 respectively). ABCA1 was suggestive of a PB>50%, with high mRNA levels in CHD patients respect HS (*p* = 0.001) while border significances were obtained between PB>50% and PB<50% (*p* = 0.052) and between PB<50% and HS (*p* = 0.065) (**Fig 5K** and **5L** and **Table 4**).

The univariate multinomial logistic regression analysis to predict coronary plaque features showed that SREBF2gene expression was able to predict/discriminate CPV<50 vs CPV>50 (OR = 0.702 CI 95%0.513–0.961; *p* = 0.027), NCPV<50 vs NCPV>50 (OR = 0.574 CI 95%0.391–0.845;*p* = 0.005) and TPV<100 vs TPV >100 (OR = 0.648 CI 95% 0.461–0.909; *p* = 0.012). Furthermore, ABCA1 gene expression was found to be associated with NCPV (OR_NCPV<50vsNCPV>50_ = 0.706 CI 95% 0.549–0.908; *p =* 0.007) and TPV (OR_TPV<100vsTPV>100_ = 0.784 CI 95% 0.624–0.984; *p* = 0.036) (**Table 5**). Adjusted multivariate analysis confirmed SREBF2 as independent predictor of CPV, NCP and TPV (*p* = 0.022; *p* = 0.002 and *p* = 0.006, respectively) and ABCA1 as independent predictor of NCPV and TPV (*p* = 0.002 and *p* = 0.013) (**Table 6**).

**Table 5:** Univariate multinomial logistic regression analysis to predict coronary plaque features.

	OR (95% CI)	*p* value	OR (95% CI)	*p* value	OR (95% CI)	*p* value
**Predictors**	**CPV<50 vs HS**		**CPV>50 vs HS**		**CPV<50 vs CPV>50**	
LDLR promoter methylation	1.922 (0.892–4.139)	0.095	2.447 (1.135–5.274)	**0.022**	1.273 (0.958–1.693)	0.096
LDLR gene expression	0.660 (0.434–1.003)	0.051	0.660 (0.430–1.011)	0.056	1 (0.730–1.369)	0.998
SREBF2 gene expression	0.881 (0.699–1.109)	0.280	0.618 (0.447–0.855)	**0.004**	0.702 (0.513–0.961)	**0.027**
ABCA1 gene expression	0.851 (0.706–1.025)	0.089	0.735 (0.588–0.917)	**0.007**	0.863 (0.698–1.068)	0.175
**Predictors**	**NCPV<50 vs HS**		**NCPV>50 vs HS**		**NCPV<50 vs NCPV>50**	
LDLR promoter methylation	1.940 (0.903–4.169)	0.089	2.440 (1.133–5.257)	**0.023**	1.258 (0.953–1.659)	0.105
LDLR gene expression	0.710 (0.467–1.080)	0.109	0.598 (0.383–0.932)	**0.023**	0.842 (0.598–1.185)	0.324
SREBF2 gene expression	0.910 (0.723–1.144)	0.418	0.522 (0.352–0.775)	**0.001**	0.574 (0.391–0.845)	**0.005**
ABCA1 gene expression	0.895 (0.746–1.073)	0.231	0.632 (0.485–0.823)	**0.001**	0.706 (0.549–0.908)	**0.007**
**Predictors**	**TPV<100 vs HS**		**TPV>100 vs HS**		**TPV<100 vs TPV>100**	
LDLR promoter methylation	1.893 (0.876–4.093)	0.105	2.484 (1.149–5.372)	**0.021**	1.312 (0.976–1.764)	0.072
LDLR gene expression	0.675 (0.444–1.0124)	0.065	0.642 (0.418–0.987)	**0.043**	0.952 (0.695–1.306)	0.762
SREBF2 gene expression	0.896 (0.712–1.128)	0.351	0.580 (0.409–0.823)	**0.002**	0.648 (0.461–0.909)	**0.012**
ABCA1 gene expression	0.876 (0.729–1.053)	0.159	0.687 (0.541–0.872)	**0.002**	0.784 (0.624–0.984)	**0.036**

Bold values were considered statistically significant with a p < 0.05.ABCA1, ATP binding cassette subfamily A member; CHD, coronary heart disease; CI, confidence interval; CPV, calcified plaque volume; LDLR, Low density lipoprotein receptor; NCPV, non-calcified plaque volume; OR, odds ratio; SREBF2, sterol regulatory element-binding factor 2; TPV, total plaque volume.

**Table 6:** Multivariate multinomial logistic regression analysis§ of risk factors associated with coronary plaque features.

**Predictors**	**CPV<50 vs HS**		**CPV>50 vs HS**		**CPV<50 vs CPV>50**	
**Model 1**	**OR (95% CI)**	***p* value**	**OR (95% CI)**	***p* value**	**OR (95% CI)**	***p* value**
Male	0.266 (0.078–0.909)	**0.035**	0.080 (0.019–0.343)	**0.001**	0.302 (0.082–1.116)	0.073
Dyslipidemia treatment	0.164 (0.040–0.677)	**0.012**	0.105 (0.023–0.0476)	**0.003**	0.639 (0.208–1.964)	0.434
Physical activity	0.162 (0.045–0.579)	**0.005**	0.626 (0.145–2.709)	0.531	3.878 (1.220–12.322)	**0.022**
SREBF2 gene expression	0.867 (0.651–1.156)	0.331	0.574 (0.390–0.846)	**0.005**	0.662 (0.466–0.942)	**0.022**
	**NCPV<50 vs HS**		**NCPV>50 vs HS**		**NCPV<50 vs NCPV>50**	
**Model 1**	**OR (95% CI)**	***p* value**	**OR (95% CI)**	***p* value**	**OR (95% CI)**	***p* value**
Male	0.365 (0.108–1.238)	0.106	0.24 (0.004–0.156)	**<0.001**	0.067 (0.012–0.375)	**0.002**
Dyslipidemia treatment	0.150 (0.038–0.588)	**0.007**	0.140 (0.27–0.713)	**0.018**	0.934 (0.269–3.246)	0.914
Physical activity	0.174 (0.49–0.620)	**0.007**	0.626 (0.134–2.929)	0.552	3.603 (1.010–12.851)	**0.048**
SREBF2 gene expression	0.900 (0.677–1.195)	0.466	0.441 (0.274–0.710)	**0.001**	0.490 (0.313–0.766)	**0.002**
**Model 2**	**OR (95% CI)**	***p* value**	**OR (95% CI)**	***p* value**	**OR (95% CI)**	***p* value**
Male	0.328 (0.092–1.174)	0.087	0.016 (0.002–0.115)	**<0.001**	0.049 (0.008–0.299)	**0.001**
Dyslipidemia treatment	0.165 (0.043–0.639)	**0.009**	0.161 (0.032–0.820)	**0.028**	0.974 (0.276–3.430)	0.967
Physical activity	0.201 (0.054–0.745)	**0.016**	0.790 (0.166–3.770)	0.768	3.926 (1.082–14.247)	**0.038**
ABCA1 gene expression	0.899 (0.715–1.129)	0.359	0.537 (0.379–0.762)	**<0.001**	0.598 (0.435–0.822)	**0.002**
	**TPV<100 vs HS**		**TPV>100 vs HS**		**TPV<100 vs TPV>100**	
**Model 1**	**OR (95% CI)**	***p* value**	**OR (95% CI)**	***p* value**	**OR (95% CI)**	***p* value**
Male	0.263 (0.076–0.913)	**0.035**	0.084 (0.020–0.348)	**0.001**	0.320 (0.087–1.182)	0.087
Dyslipidemia treatment	0.126 (0.031–0.516)	**0.004**	0.162 (0.036–0.731)	**0.018**	1.291 (0.408–4.079)	0.664
Physical activity	0.144 (0.039–0.531)	**0.004**	0.666 (0.154–2.875)	0.586	4.638 (1.412–15.239)	**0.011**
SREBF2 gene expression	0.900 (0.673–1.204)	0.478	0.525 (0.348–0.794)	**0.002**	0.584 (0.397–0.858)	**0.006**
**Model 2**	**OR (95% CI)**	***p* value**	**OR (95% CI)**	***p* value**	**OR (95% CI)**	***p* value**
Male	0.236 (0.066–0.850)	**0.027**	0.066 (0.015–0.284)	**<0.001**	0.278 (0.074–1.039)	0.057
Dyslipidemia treatment	0.142 (0.035–0.575)	**0.006**	0.184 (0.041–0.824)	**0.027**	1.296 (0.411–4.084)	0.658
Physical activity	0.171 (0.045–0.652)	**0.010**	0.812 (0.186–3.546)	0.781	4.741 (1.443–15.577)	**0.010**
ABCA1 gene expression	0.884 (0.703–1.113)	0.295	0.638 (0.479–0.848)	**0.002**	0.721 (0.558–0.932)	**0.013**

Bold values were considered statistically significant with a p < 0.05.

^§^Multivariate analysis corrected for male gender.

ABCA1, ATP binding cassette subfamily A member; CHD, coronary heart disease; CI, confidence interval; CPV, calcified plaque volume; NCPV, non-calcified plaque volume; OR, odds ratio; SREBF2, sterol regulatory element-binding factor 2; TPV, total plaque volume.

## Discussion

Here, we evaluated in PBMNCs from suspected CHD patients and HS, undergoing CCT, the methylation status of specific regulatory regions in principal genes involved in cholesterol pathway. Furthermore, we have analyzed the mRNA levels of the selected genes.

Some studies associated mutations or polymorphisms in LDLR gene and impairment of its function with atherosclerosis burden and/or an increased incidence of CVDs and a high risk for both incident and recurrent cardiac acute events [37–40]. In particular, Ten Kate et al. performed an association study considering the mutational status of LDLR gene and the extent of subclinical coronary atherosclerosis, detected by CCT, expressed in terms of Diseased Segment Score (DSS) [40].

Our study investigated for the first time the methylation and expression levels of LDLR with quantitative features derived from CCT. A study by Liu et al., suggested that abnormal lipid homeostasis may contribute to the pathogenesis of vascular calcification. Indeed, authors performed an immunohistochemical analysis reporting an up-regulation of LDLR in atherosclerotic vascular tissues and a parallel increase in calcified plaque deposition [41]. Even if LDLR expression was evaluated at protein level and in vascular tissues, our results, obtained from PBMNCs, are in line with the above-mentioned data. We established in a non-invasive way that high levels of LDLR gene were associated with the presence of CHD and in particular to obstructive CHD. Furthermore, the degree of methylation in a promoter region of the same gene was more associated with severe CACS.

SREBF2 performs a fine control on the transcription of sterol-regulated genes such as LDLR and ABCA1. Previous studies reported an altered SREBF2 gene expression in atherosclerotic tissues as well as an association of specific genotypes with the risk of cardiovascular events and sudden cardiac death [42–45]. Shchelkunova et al., analyzed the expression levels of SREBF2 gene in human aortic samples. Results showed that SREBF2 mRNA was found comparable to non-diseased tissue in initial lesions. Otherwise SREBF2 transcript was found to be progressively up-regulated in fatty streaks and fibrous lipid plaques, supporting an active role of this biomolecule during the atherosclerosis progression [46]. The present study shows an association between the circulating expression levels of SREBF2 mRNA in PBMNCs and the presence of obstructive CHD. We have detected, in non-invasive manner, that SREBF2 gene expression was a predictor of critical stenosis independent by gender, so it could be a surrogate biomarker of CHD and disease severity.

Regarding the molecular analysis of ABCA1, literature data reported significant associations of dysregulation of this cholesterol transporter with the atherosclerotic process and CHD [47–52]. Indeed, an involvement of ABCA1 has been demonstrated in the formation and composition of carotid atherosclerotic plaques with an upregulation of ABCA1 levels between atherosclerotic and healthy tissues [51], as well as the significance of this biomarker in plaque rupture [52]. In our study we have shown from peripheral blood sample that ABCA1 mRNA was up-regulated in patients with CHD underwent to CCT, suggesting a possible implication in disease diagnosis and high-risk plaque detection. In light of our results, we hypothesize that ABCA1 could be a predictive biomarker of CHD while SREBF2 could be involved in the identification of obstructive stenosis. In addition, plaque feature results suggest SREBF2 gene expression as predictive of total plaque burden (both calcified and non-calcified plaque component) while ABCA1 could be related to non-calcified plaque composition hence allowing to identify high-risk plaque.

Considering that the selected molecular markers are all involved in the cholesterol metabolism, we firstly compared total, LDL, HDL, and cholesterol serum concentrations between CHD and HS group, reporting no significant alterations at the time of CCT. Following, in order to investigate the effects of dyslipidemia treatments on the expression of the selected molecular markers, we performed a subgroup analysis comparing treated vs untreated subjects, reporting no effects of medications on ABCA1 and SREBF2 gene expression and on the percentage of LDLR promoter methylation.

The present study has some limitations. We recognize that a genome-wide approach could have been the best strategy in identifying more molecular markers associated to CHD presence, severity and coronary plaque features. The epigenetic and analysis was performed considering only the methylation levels of limited portions in promoters and regulatory elements in a specific set of genes of interest already recognized to be related to atherogenesis and endothelial function in vascular tissues. Although GWAS and EWAS require huge amount of blood samples, further studies are needed to evaluate other epigenetic modified sequences and we cannot exclude that others post transcriptional events (i.e. microRNAs) may influence mRNA expression levels.

Another limitation is represented by the small sample size, moreover our analysis was conducted only in suspected CHD patients but an evaluation of patients with known CHD should be considered for future analysis. It could be of great interest to evaluate a possible association of these epigenetic-sensitive biomarkers with occurrence/recurrence of cardiac acute events. At the same time, a further analysis could investigate the association of such biomarkers with myocardial ischemia to assess the morphological-functional relationship in CHD patients.

Moreover, the prognostic value of the studied cholesterol/metabolic biomarkers for cardiovascular events and their contribution to the risk-stratification of the population undergoing diagnostic CCT could be tested in a larger cohort with prospective follow-up.

Finally, although the increasing technological widespread, the costs incurred to perform gene expression/methylation analysis have to be taken into account, although the high sensitivity, specificity and reproducibility of qRT-PCR technique.

## Conclusions and future perspectives

Our study could have an important clinical impact, representing a possible initial epigenetic/molecular-sensitive approach in the diagnosis and prediction of CHD through a non-invasive approach. Therefore, the evaluation of reproducible and non-invasive techniques for assessing endothelial function should enable screening of large populations and may guide interventions designed specifically to reduce the individual vascular risk.

## Supporting information

S1 Appendix Supplementary methods. (DOCX)

S1 Table Primers used for quantitative realtime PCR. (DOCX)

S1 Fig CHD molecular features in relationship with CACS levels. **(A)** % of LDLR promoter methylation/total input in CHD patients categorized according to CACS levels in: Normal (CACS = 0) (n = 32), Low (CACS = 1–100) (n = 21), Moderate (CACS = 101–400) (n = 18), and Severe (CACS>400) (n = 24). (**B)** SREBF2 mRNA relative expression in CHD patients categorized according to CACS levels; (**C)** ABCA1 gene relative expression in CHD patients categorized according to CACS levels.
(TIF)

## Abbreviations

ABCA1: ATP binding cassette subfamily A member 1
CCT: cardiac computed tomography
CHD: coronary heart disease viruses
CPV: calcified plaque volume
LDLR: low density lipoprotein receptor
NCPV: non-calcified plaque volume
PB: plaque burden
SREBF2: sterol regulatory element-binding factor 2
TPV: total plaque volume
