# Supplementary material for: Evidence of association of circulating epigenetic-sensitive biomarkers with suspected coronary heart disease evaluated by Cardiac Computed Tomography
Source: PLoS One. 2019 Jan 23;14(1):e0210909. doi: 10.1371/journal.pone.0210909 (PMC6343931; doi:10.1371/journal.pone.0210909)
Supplement: S1 Appendix — (DOCX) [file pone.0210909.s001.docx]

**Evidence of association of a circulating epigenetic-sensitive biomarker with suspected coronary heart disease evaluated by Cardiac Computed Tomography**

Teresa Infante, Ernesto Forte, Concetta Schiano, Bruna Punzo, Filippo Cademartiri, Carlo Cavaliere, Marco Salvatore, Claudio Napoli

**SUPPORTING INFORMATION**

**Supplementary Methods**

**Study population**

All clinical characteristics such as laboratory parameters, presence of cardiovascular risk factors, and medical history were accurately recorded.

Dyslipidemia was defined as treatment with drugs or fasting serum total cholesterol ≥240 mg/dL, or LDL cholesterol ≥140 mg/dL, or high-density lipoprotein cholesterol <40 mg/dL, or triglyceride ≥150 mg/dL. Diabetes was defined as treatment with drugs or fasting blood glucose ≥126 mg/dL. Hypertension was defined as treatment with drugs or systolic blood pressure (SBP) ≥140 mmHg or diastolic blood pressure (DBP) ≥90 mmHg. Anthropometrical measurements including body weight and height were recorded and body mass index (BMI) was calculated. Blood pressure and resting heart rate were measured after ≥5 min rest with a sphygmomanometer. Physical activity in HS and CHD patients was evaluated according to the current WHO guideline [1]. None of the recruited subjects had physical disabilities. All patients in treatment for dyslipidemia were affected by polygenic dyslipidemia not on a familial genetic basis.

**Sample collection and molecular analysis**

From all the study participants peripheral venous blood samples were collected in EDTA tubes after 6-8 hours fasting. All tubes were centrifuged at 1900 x g for 10 minutes at 4°C within 1 hour of collection to separate plasma and cellular components.

**Methylated DNA Immunoprecipitation (MeDIP)**

Cells were resuspended in genomic digestion buffer with proteinase K and incubated at 50°C over night. Genomic DNA was extracted with phenol/chloroform/isoamyl alcohol and 30 µg were sheared (10 cycles, 15 s “ON”, 15 s “OFF” at 20% of amplitude) in fragments between 100-800 bp using the Q125 sonicator (Qsonica, USA). Shared DNA was analyzed on agarose gel. MeDIP was performed using α-5′methyl-cytosine antibody; samples were rotated overnight at 4°C in the presence of magnetic beads. The 10% of IP incubation mix of each DNA shared sample was stored as input for the comparison with immunoprecipitated DNA. After washes, immunoprecipitated DNA was eluted in TE buffer. The amount of methylated DNA enrichment in MeDIP samples compared to the respective INPUT sample was detected by qRT-PCR CFX96 Touch Real-Time PCR Detection System (BioRad Laboratories, Ltd, USA) with iQ SYBR® Green Supermix (BioRad Laboratories, Ltd, USA). A set of specific primer pairs provided in the kit and targeting specific DNA sequences were used for checking MEDIP efficiency. The Methyl DNAIP controls revealed IP efficiency. qRT-PCR data were expressed as percentage of methylated DNA IP compared to input (% of DNAIP/total input). Each sample was analyzed in triplicate and data expressed as mean **±** standard error.

**Methylation prediction analysis**

For the identification and prediction of CpG islands on regulative genomic elements were considered the following regions:

1. ***LDLR* (Ref Seq: NM_001195802):**
   - a promoter region located from -317bp to -142 bp the TSS (chr19:11089234+11089409);
   - an intron1 region located from +880 bp to +1092 bp the TSS (chr19:11090431+11090643) and matching with a 767bp CpG island (chr19:11090244+11091010);
2. ***SREBF2* (RefSeq: NM_004599):**
   - a promoter region located from -717 bp to -550 bp the TSS (chr22:41832553+41832720) and matching with a 1477 bp CpG island (chr22:41832504+41833980);
3. ***ABCA1* (RefSeq: NM_005502):**
   - a promoter region located from -24.782 bp to -24.633 bp the TSS (chr9:104928257+104928406) and matching with a 951 bp CpG island (chr9:104927567-104928517).

**RNA extraction and quantitative realtime PCR assay**

RNA quantity and quality were determined using a NanoDrop ND-1000 spectrophotometer (Thermo Fischer Scientific, USA). RNA (500ng) was reverse transcribed with SuperScript® III First-Strand Synthesis System for RT-PCR (Thermo Fischer Scientific, USA) according to the manufacturer’s instructions in 20μL reaction. The relative expression levels of mRNA were measured by CFX96 Touch Real-Time PCR Detection System (BioRad Laboratories, Ltd, USA) using iQ SYBR® Green Supermix (BioRad Laboratories, Ltd, USA) and 300nM each primer pair. Primers were designed by Primer 3 software (<http://bioinfo.ut.ee/primer3-0.4.0/>) and synthesized by Life Technologies. The specificity of each oligonucleotide pair was verified with the BLAST program. Melt curve analysis was performed to verify a single product species.

**Imaging protocol**

A prospectively ECG-triggered high pitch spiral acquisition (FLASH) without contrast medium was performed for calcium score evaluation (slice thickness of 3mm, increment of 3 mm, small FOV). Afterwards, patients underwent angiographic Cardiac CT scans with IV contrast material (50 mL@5ml/s of iodinated contrast agent - Iomeprol 400 mg I/ml - Iomeron 400 (Bracco, Italy), followed by 50mL@5ml/s of saline flush; scans were performed with retrospective ECG gating and with prospective ECG-tube current modulation (window 25%-75% of the R-R interval). Automated attenuation-based anatomical tube current (mAs) modulation (CARE Dose4D, Siemens) and automated attenuation-based tube voltage (kV) selection functionality (CARE kV, Siemens) were used for dose reduction and optimization. Data were reconstructed with a dedicated 3rd generation advanced modeled iterative reconstruction (ADMIRE, Siemens) with a strength level of 3 using different convolution kernels (Bv36, Bv40, Bv44 and Bv49) at best diastolic and best systolic phases (slice thickness of 0.75mm, increment of 0.4mm, pixel matrix size of 512x512) with the smallest FOV possible. Images were analyzed on an offline dedicated workstation (Syngo.Via VB10B, Siemens) where multiplanar reformations (MPR), maximum intensity projections (MIP), curved multiplanar reformations (c-MPR) and 3D volume rendering images were generated.

For calcified plaque composition analysis, patients were categorized into four groups based on their Agatston score according to a modified version of Rumberger’s Mayo Clinic guidelines [2] as follow: normal (CACS=0), low (CACS=1-100),moderate (CACS=101–400) and severe (CACS>400). For CPV, NCPV,TPV and PB, patients were grouped considering the median value as threshold [3].

**References**

1. WHO Guidelines Approved by the Guidelines Review Committee. Global Recommendations on Physical Activity for Health. Geneva: World Health Organization; 2010. PMID:26180873.

2. Rumberger JA, Brundage BH, Rader DJ, Kondos G. Electron beam computed tomographic coronary calcium scanning: a review and guidelines for use in asymptomatic persons.Mayo Clin Proc. 1999;74(3):243-52. <https://doi.org/10.4065/74.3.243> PMID:10089993.

3. [Clark D 3rd](https://www.ncbi.nlm.nih.gov/pubmed/?term=Clark%20D%203rd%5BAuthor%5D&cauthor=true&cauthor_uid=29688308), [Nicholls SJ](https://www.ncbi.nlm.nih.gov/pubmed/?term=Nicholls%20SJ%5BAuthor%5D&cauthor=true&cauthor_uid=29688308), [St John J](https://www.ncbi.nlm.nih.gov/pubmed/?term=St%20John%20J%5BAuthor%5D&cauthor=true&cauthor_uid=29688308), [Elshazly MB](https://www.ncbi.nlm.nih.gov/pubmed/?term=Elshazly%20MB%5BAuthor%5D&cauthor=true&cauthor_uid=29688308), [Kapadia SR](https://www.ncbi.nlm.nih.gov/pubmed/?term=Kapadia%20SR%5BAuthor%5D&cauthor=true&cauthor_uid=29688308), [Tuzcu EM](https://www.ncbi.nlm.nih.gov/pubmed/?term=Tuzcu%20EM%5BAuthor%5D&cauthor=true&cauthor_uid=29688308), [Nissen SE](https://www.ncbi.nlm.nih.gov/pubmed/?term=Nissen%20SE%5BAuthor%5D&cauthor=true&cauthor_uid=29688308), [Puri R](https://www.ncbi.nlm.nih.gov/pubmed/?term=Puri%20R%5BAuthor%5D&cauthor=true&cauthor_uid=29688308). Visit-to-visit cholesterol variability correlates with coronary atheroma progression and clinical outcomes. [Eur Heart J](https://www.ncbi.nlm.nih.gov/pubmed/?term=Visit-to-visit+cholesterol+variability+correlates+with+coronary+atheroma+progression+and+clinical+outcomes). 2018;39(27):2551-58. <https://doi.org/10.1093/eurheartj/ehy209> PMID:29688308.
