# Supplementary material for: Evidence of association of circulating epigenetic-sensitive biomarkers with suspected coronary heart disease evaluated by Cardiac Computed Tomography
Source: PLoS One. 2019 Jan 23;14(1):e0210909. doi: 10.1371/journal.pone.0210909 (PMC6343931; doi:10.1371/journal.pone.0210909)
Supplement: S1 Table — (DOCX) [file pone.0210909.s002.docx]

**S1 Table. Primers used for quantitative realtime PCR**

| **Genomic regulative elements** | **Forward primer** | **Reverse primer** |
| --- | --- | --- |
| ABCA1 5’UTR | CCCGGCCTCTGTTTATGTAG | ACACCTGCTGTACCCTCCAC |
| LDL receptor promoter | TCTTCACCGGAGACCCAAAT | GAGTTTGCAGTGGGGTGATT |
| LDL receptor intr1 | CCGAATTCCATTGGGTGTAG | GTCGCCTTTGTGACAGGAAC |
| SREBF2 promoter | CTGGGACAATCTGCAACCTT | GTCATCTTCCCAGCTGCCTA |
| **Gene expression** | **Forward primer** | **Reverse primer** |
| RPS18 | CGATGGGCGGCGGAAAATA | CTGCTTTCCTCAACACCACA |
| ABCA1 | GTCCTCTTTCCCGCATTATCTGG | AGTTCCTGGAAGGTCTTGTTCAC |
| LDL receptor | CAGATATCATCAACGAAGC | CCTCTCACACCAGTTCACTCC |
| SREBF2 | AGGAGAACATGGTGCTGA | TAAAGGAGAGGCACAGGA |
