# Supplementary figures and images for: Evidence of association of circulating epigenetic-sensitive biomarkers with suspected coronary heart disease evaluated by Cardiac Computed Tomography
Source: PLoS One. 2019 Jan 23;14(1):e0210909. doi: 10.1371/journal.pone.0210909 (PMC6343931; doi:10.1371/journal.pone.0210909)

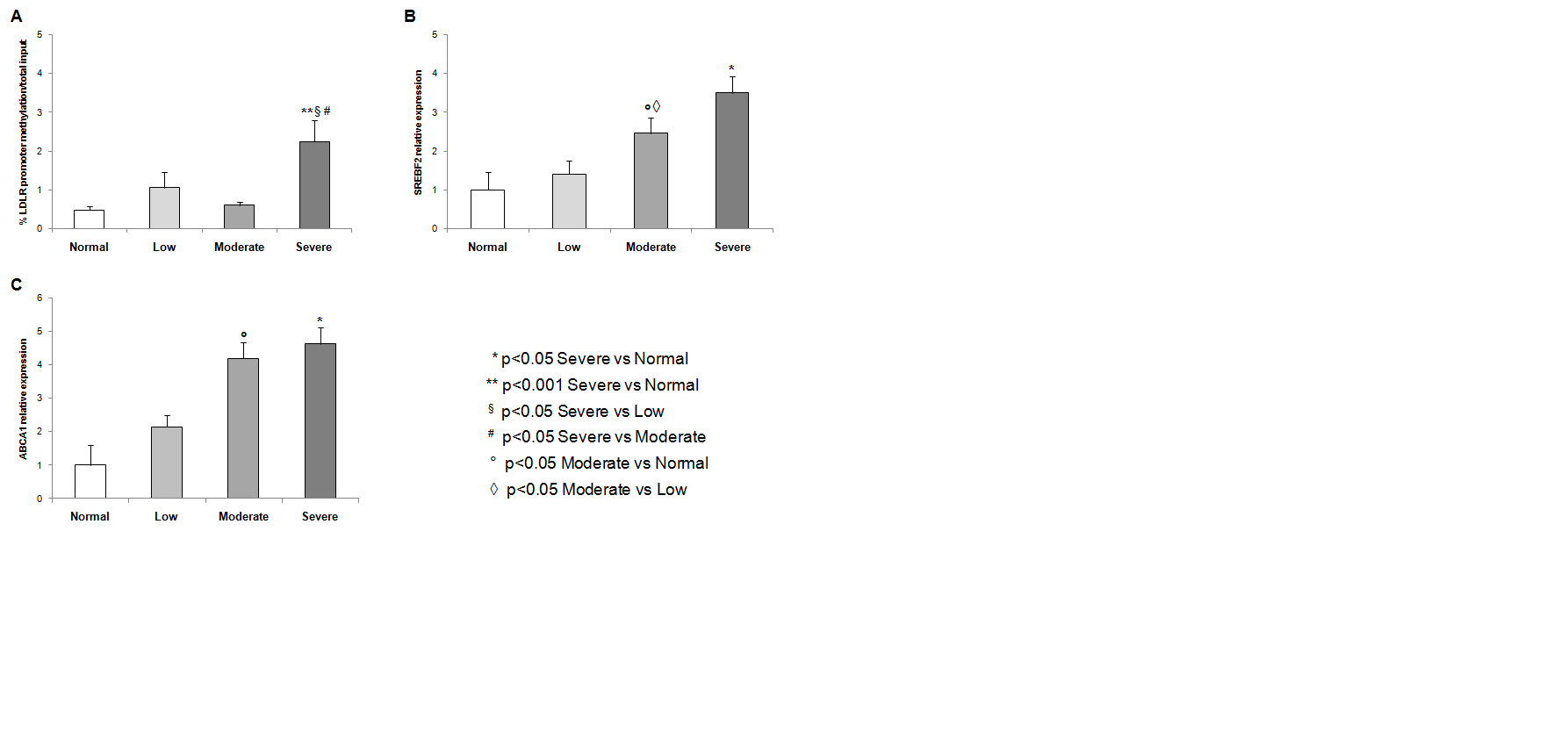

Supplement: S1 Fig — (A) % of LDLR promoter methylation/total input in CHD patients categorized according to CACS levels in: Normal (CACS = 0) (n = 32), Low (CACS = 1–100) (n = 21), Moderate (CACS = 101–400) (n = 18), and Severe (CACS>400) (n = 24). (B) SREBF2 mRNA relative expression in CHD patients categorized according to CACS levels; (C) ABCA1 gene relative expression in CHD patients categorized according to CACS levels. (TIF) [file pone.0210909.s003.tif]
